# Comparative Biochemical and Pharmacodynamic Analyses of *Asarum heterotropoides* Fr. Schmidt var. Mandshuricum (Maxim) Kitag and *Asarum sieboldii* Miq var. Seoulense Nakai Roots

**DOI:** 10.3390/ph17101301

**Published:** 2024-09-30

**Authors:** Huiling Li, Zhiqing Wang, Guangyuan Zhao, Yanhong Wang, Xuanwei Xu, Yingping Wang, Ze Zhang, Guanghui Wang

**Affiliations:** 1 College of Chinese Medicinal Materials, Jilin Agricultural University, Changchun 130118, China; lhl6162023@163.com (H.L.); wguanghui1020@163.com (G.W.); 2 Ginseng and Antler Product Quality and Safety Risk Assessment Laboratory, Ministry of Agriculture and Rural Affairs, Jilin Agricultural University, Changchun 130118, China; yanhong-w@163.com (Y.W.); xuanweix@jlau.edu.cn (X.X.); 3 State & Local Joint Engineering Research Center of Ginseng Breeding and Application, College of Chinese Medicinal Materials, Jilin Agricultural University, Changchun 130118, China

**Keywords:** anti-inflammatory, asarinin, essential oil composition, ethanol extract, ear oedema test, LC-MS, GC-MS, xixin

## Abstract

**Background:***Asarum heterotropoides* and *Asarum sieboldii* are commonly used in traditional Chinese medicine. However, little is known about how they differ in terms of essential oil (EO) and ethanol extract (EE) content and composition. Moreover, the effect of various geographical locations on the essential oil (EO), ethanol extract (EE), and asarinin content of different *Asarum* samples remains unknown. We tested four root-drying methods, i.e., soil removal and shade drying (P1), water washing and shade drying (P2), and water washing and drying at 30 °C (P3) and 40 °C (P4). We used LC-MS and GC-MS to investigate these differences. We also investigated the pharmacodynamic effects of EO and EE. **Results**: Overall, the EO, EE and asarinin contents of the analysed samples were 19.21–51.53 μL.g^−1^, 20.00–45.00 μL.g^−1^, and 1.268–2.591 mg.g^−1^, respectively. P1 treatment yielded the lowest volatile oil content compared to the other three treatments. GC-MS analysis revealed 78 EO components. Among the six major EO components, eucarvone, 3,5-dimethoxytoluene, and methyl eugenol were higher in *A. heterotropoides* than in *A. sieboldii*. However, the latter had a higher myristicin content. LC-MS analysis identified 888 EE components in roots and leaves of *A. heterotropoides* and *A. sieboldii*; 317 differentially accumulated metabolites were identified. EO and EE showed a dose-dependent reduction in the degree of swelling and an increase in the inhibition rate of drug concentration on acetic acid writhing in mice. *Asarum* EO proved to be more effective than EE in the pharmacodynamic study. **Conclusions**: We conclude that *Asarum* species show inter- and intra-specific differences in EO and EE content and composition, which may influence the pharmacodynamics of *Asarum* root extracts.

## 1. Background

*Asarum* originates from Asia, but its members are naturally distributed throughout the temperate zones of the northern hemisphere. Among the members of the genus *Asarum*, *Asarum heterotropoides* Fr. Schmidt var. mandshuricum (Maxim) Kitag and *Asarum sieboldii* Miq var. seoulense Nakai are of particular importance because of their frequent use in traditional Chinese medicine (TCM) [1,2]. 

The roots of both species are listed as “xixin” in the *Chinese Pharmacopoeia*. Xixin has anti-inflammatory, anticancer, antibacterial, and analgesic effects [3], and is commonly used in TCM to treat colds, headaches, toothaches, rhinorrhoea, wheezing, inflammation, and others [4]. Apart from its use in TCM, *Asarum* essential oils (EOs) have been reported to have activities against phytopathogens and insects [5,6]. The health-promoting properties and activities against insects and pests of crops are due to the composition of their metabolomes, in particular, the EOs, monoterpenes, lignans, alkaloids, alcohols, asarinin, and amides [7,8]. Asarinin from *Asarum* species has strong bioactivity such as anticancer [9], antihypertensive, and antiangiogenic activities [10]. Studies on the composition of *Asarum* oil have shown that methyl eugenol, sesamin, safrole, N-isobutyl-(2E,4Z,8Z,10E)-dodecatetraenamide, pentadecane eucarvone, 3,5-dimethoxytoluene, safrole, myristicin, and elemicin are present in higher percentages [11,12]. Meanwhile, the ethanolic extracts consist of lignans, monoterpenes [11], phenanthrene derivatives, isobutyle amides, and pehnylpropanoids [13,14]. However, the content and composition have been reported to be influenced by extraction methods, species and cultivar, growing conditions, and geography [11,14]. 

Within China, *Asarum* is a particularly famous herb in the northeast of the country, such as Xinbin and surrounding areas in Liaoning Province, and in eastern mountainous areas such as Tonghua County, Liuhe County, Ji’an County, and Fusong County in Jilin Province. As the main growing area, wild *Asarum* is mainly found in the humid and fertile understorey of the Changbai Mountains or in mid-mountainous areas [14,15]. Such a wide area highlights the need to investigate how the same cultivar grown in different areas would affect the EO content (EOC) and composition of ethanol extracts (EEs). Previous work in this regard on China has reported the presence of 48–77 volatile oil compounds. The authors also reported the presence of significant variation in the active constituents of *A. heterotropoides* var. mandshuricum [14]. Therefore, *Asarum* grown in different areas needs to be further investigated for the effect of area of cultivation on the content of active ingredients. In addition to the area of cultivation, the way in which the *Asarum* roots are processed prior to extraction of volatiles or EOs is one of the most important factors. It has been shown for several medicinal plant species that the way the roots (or other useful tissues) are processed can influence the yield of EOs. For example, an increase in menthone and pulegone and a decrease in limonene was observed when aerial parts of Moroccan *Mentha pulegium* L. were dried compared to fresh samples [16]. Similarly, different drying methods were found to affect phenol, total flavonoids, liquiritin, and *glycyrrhizic* acid in *Glycyrrhiza uralensis* (liquorice) [17]. Thus, processing of *Asarum* roots may or may not lead to changes in the content of EOs, ethanolic extracts, and asarinin. In addition, most of the literature on EO, EE, and asarinin content (AsC) showed that there are differences within species. However, limited work has highlighted the differences between *A. heterotropoides* and *A. sieboldii*.

The objective of this study was to compare the effect of four different *Asarum* root-drying methods on EO, EE, and AsC. We also aimed to compare the EE as well as metabolome profiles of the *A. heterotropoides* and *A. sieboldii* samples. Finally, we tested the pharmacodynamic effects of *Asarum* volatile oil and EE. For these objectives, first, we determined the effect of four different processing methods, i.e., soil + shade drying (P1), water washing + shade drying (P2), water washing + drying at 30 °C (P3), and water washing + drying at 40 °C (P4), on EOC, EE content (EEC), and AsC. We then determined the EO and EE composition of 36 root samples. After that, we investigated the differential metabolomic profiles of both species. Finally, we investigated the anti-inflammatory and analgesic effects of extracts from both species. Our results are valuable for TCM (and the pharmaceutical industry) as well as for *Asarum* growers to choose the appropriate processing method for *Asarum* drying.

## 2. Results

### 2.1. Effect of Asarum Root-Drying Methods on Biochemical Content Yield

**Figure 1:**
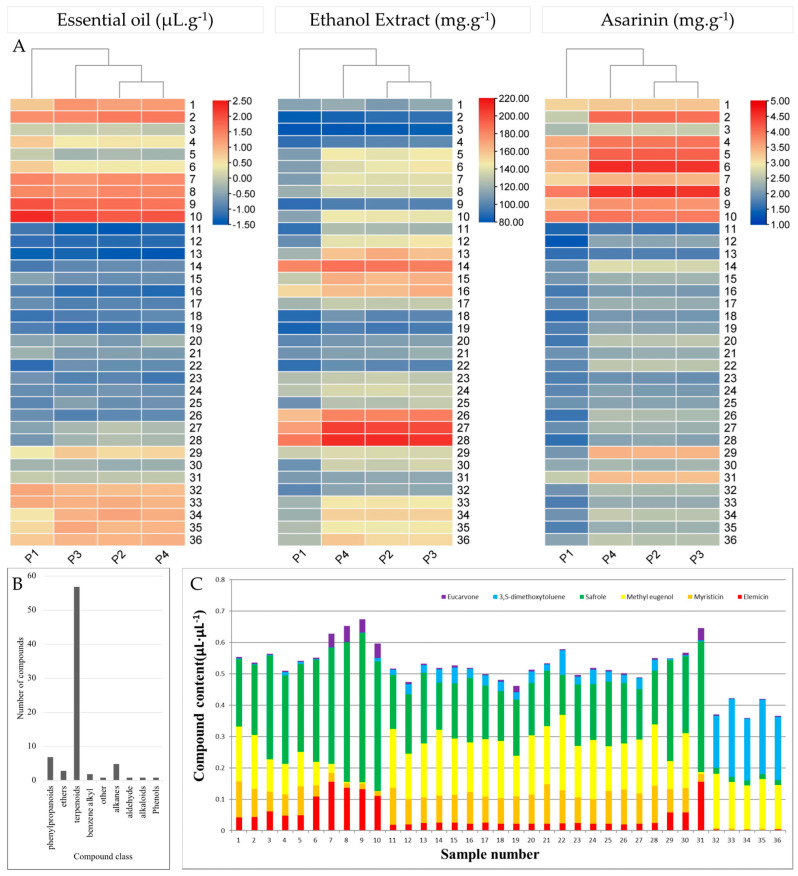
(**A**) Essential oil, ethanol extract, and asarinin content in *Asarum* roots. (**B**) Number of metabolites accumulated in *Assarum* roots according to class. (**C**) Content distribution of six compounds in sample essential oil in 36 *Asarum* samples (roots). Samples 1–31 are *Asarum heterotropoides* Fr. var. mandshuricum (Maxim.) Kitag (cultivated), and Samples 32–36 are *Asarum sieboldii* Miq var. seoulense Nakai (wild).

First, we investigated whether the four processing methods showed any variation in volatile oil content. To do this, we processed the *Asarum* samples by removing the soil + drying in the shade (P1), washing with water + drying in the shade (P2), washing with water + drying at 30 °C (P3), and washing with water + drying at 40 °C. The results showed that the volatile oil content of P1 was lower than the other three methods. The samples washed with water and dried in the shade or in the oven at 30 °C and 40 °C had no significant differences among the 36 *Asarum* samples (Figure 1A; Table S2). 

Overall, the EOC of the analysed samples ranged from 19.21 to 51.53 μL.g^−1^. The content of EO in 90.44% of the samples was 20.00~45.00 μL.g^−1^.

The EOC of cultivated (19.21–47.65 μL.g^−1^) and wild (30.14–51.53 μL.g^−1^) samples showed visible differences, with the latter having 8.73–25.16% higher contents. Similarly, the EOC of *Asarum* from different regions was variable, such that the highest levels were found in samples from Jian (30.10–51.53 μL.g^−1^), followed by Fusong (36.02–47.65 μL.g^−1^), Lyeyang (34.77–43.65 μL.g^−1^, Ningqiang (33.73–43.76 μL.g^−1^), Wang Qing (33.21–40.65 μL.g^−1^), Xinbin (27.69–33.95 μL.g^−1^), Tonghua (20.23–33.37 μL.g^−1^), and Liuhe (19.21–31.74 μL.g^−1^) (Table 1). These observations suggest that the place of origin of *Asarum* may be a possible factor influencing EOC (the growth years and different sources influence the EOC). 

Regarding the content of ethanolic extracts, the processing methods did not show any significant effect (Figure 1A; Table S2). In general, the EEC in *Asarum* roots was 80.18–211.06 mg.g^−1^. Overall, 83.09% of the *Asarum* samples had an EEC in the range of 90–170 mg.g^−1^, with wild (90.03–144.36 mg.g^−1^) having relatively lower contents than the cultivated varieties (80.18–211.06 mg.g^−1^). Regarding the origin of *Asarum* cultivars and wild types, the EEC content range was different (Figure 1A; Table S2), with the maximum in samples from Tonghua (91.14–211.06 mg.g^−1^) and the minimum in samples from Xinbin (105.88–136.01 mg.g^−1^) (Table 1).

The AsC in the 36 samples analysed ranged from 1.268 to 2.591 mg.g^−1^; 80.88% of the samples contained 1.500–3.500 mg.g^−1^. The effect of the processing method was similar to that of EOC and EEC, i.e., P1 had the lowest content. In general, P3 and P4 had a higher AsC. Jian (2.41–4.59 mg.g^−1^) had the highest maximum AsC, followed by Fusong (2.61–4.07 mg.g^−1^), Wangqing (1.59–3.45 mg.g^−1^), Xinbin (2.19–3.35 mg.g^−1^), Liuhe (1.27–2.79 mg.g^−1^), Lyeyang (1.77–2.57 mg.g^−1^), and Ningqiang (1.74–2.57 mg.g^−1^). 

Finally, the *A. heterotropoides* samples on average exhibited lower average EOC (3.19 vs. 3.37 μL.g^−1^), EEC (3.26 vs. 3.37 mg.g^−1^), and asarinin (3.294 vs. 3.40 mg.g^−1^) compared to *A. sieboldii* samples. However, when compared the highest content of each of EOC, EEC, and asarinin, *A. heterotropoides* samples had the maximum contents. 

These results indicate that the biochemical yield (EOC, EEC, and asarinin) differs between cultivars and wild-type *Asarum* collected from different regions. Moreover, it can be understood that washing and drying in an oven may be a useful processing method to obtain higher EOC, EEC, and asarinin.

### 2.2. Essential Oil Composition of 36 Asarum Samples

Next, we determined the EO composition of the 36 *Asarum* samples processed by the P4 method (washing with water and drying at 40 ℃) by using GC-MS analysis. A total of 78 compounds were identified (Table S3). These compounds belonged to nine compound classes (Figure 1B). The highest number of compounds were classified as terpenoids (57), followed by phenylpropanoids (07), alkanes (05), ethers (3), benzene alkyl (02), aldehyde (01), alkaloids (01), phenols (01), and others. The highest content in most of the examined samples was of methyl eugenol, 1,3-benzodioxole, 5-(2-propenyl)-, 1,3-dimethoxy-5-methylbenzene, 2,4-cycloheptadien-1-one,2,6,6-trimethyl-, and 1,2,3-trimethoxy-5-methylbenzene. Considering the origin of the samples, we observed that Fusong, Jiang, Liuhe, Tonghua, Wangqing, Xinbin, Ningqiang, and Lyeyang samples (1–2) had 61, 61, 76, 75, 49, 68, 46, and 45 metabolites. These results show that the *Asarum* samples from different regions have different EO compositions (Table S3). 

Furthermore, we quantified the six major EO components, i.e., eucarvone, 3,5-dimethoxytoluene, safrole, methyl eugenol, myristicin, and elemicin (Figure 1C). The contents of eucarvone, 3,5-dimethoxytoluene, and methyl eugenol in the EO of *A. heterotropoides* were higher than *A. sieboldii* Miq. The contents of these six EC components ranged from 48.2 to 68.4 μL.μL^−1^. In addition, the total content of these six essential components in wild samples was higher than that in cultivated samples. The most significantly higher component in wild compared to cultivated samples was elemicin, which was significantly higher. Notably, the contents of myristicin in the EO of *A. sieboldii* Miq were higher than *A. heterotropoides,* and the content of the six components is less than 45 μL.μL^−1^ (Figure 1C).

### 2.3. Comparative Metabolome Analysis of A. heterotropoides and A. sieboldii Roots and Leaves

**Figure 2:**
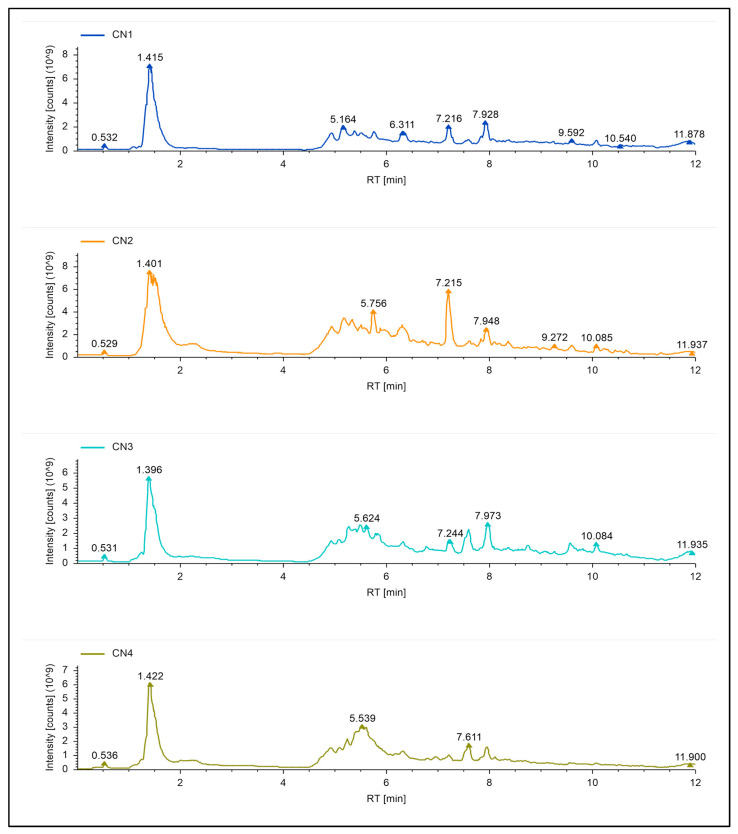
Total ion current chromatograms of ethanol extract samples. CN1 indicates L24-3, CN2 indicates L32-3, CN3 indicates R24-3, and CN4 indicates R32-3.

**Figure 3:**
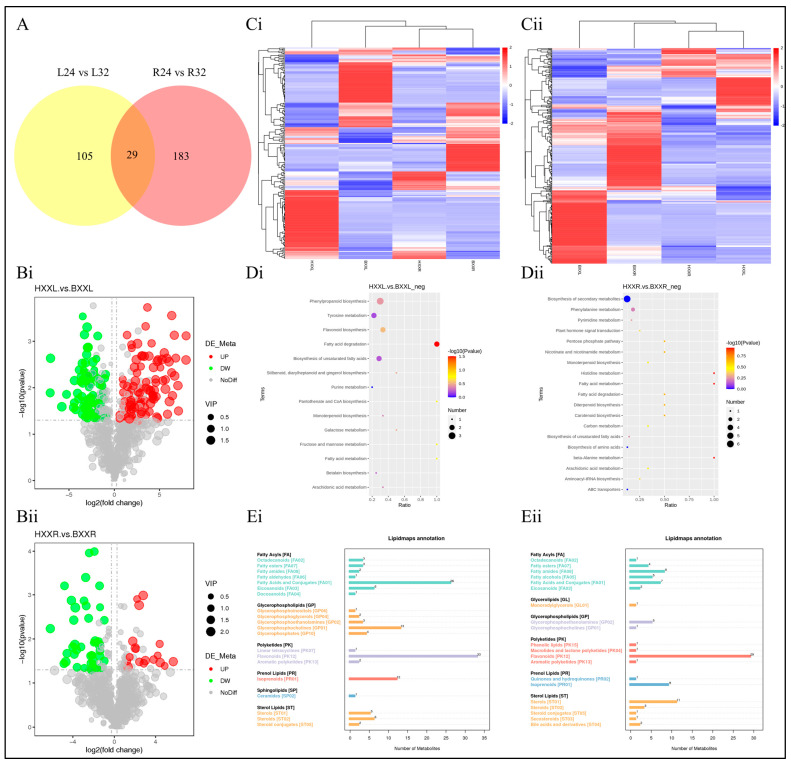
Comparative metabolome analysis of *A. heterotropoides* and *A. sieboldii* roots and leaves. (**A**) Venn diagram of common and specific metabolites in roots and leaves of the two species. (**Bi**) Volcano map of leaf vs. root differential metabolites in negative ion mode and (**Bii**) Volcano map of leaf vs. root differential metabolites in positive ion mode. The abscissa represents log2 (fold change); the ordinate represents −log10 (*p*-value); each point represents a compound; the size of each point represents the VIP value, i.e., the larger the point, the larger the VIP value; up (red) represents the compounds with significant difference and upregulation; DW (green) represents the compounds with significant difference and downregulation; no Diff (grey) represents the compounds with no significant difference; the greater the absolute value of the abscissa, the greater the difference between samples; the larger the ordinate value, the more significant the difference between the samples, and the more reliable the result. (**Ci**) Heatmap displaying the variation in differential metabolite accumulation in roots and leaves of samples, with negative and (**Cii**) with positive ion modes. The ruler indicates the level of metabolite expression. The redder the colour, the higher the metabolite expression. The bluer the colour, the lower the metabolite expression. Each row represents a metabolite. (**Di**) KEGG pathway enrichment of the differentially accumulated metabolites in leaf vs. roots, with negative and (**Dii**) with positive ion modes. (**Ei**) Annotation of the lipid compounds detected, with negative and (**Eii**) with positive ion modes. HXXL indicates L32, BXXL indicates L24-3, HXXR indicates R32, and BXXR indicates R24-3. L = leaf and R = root.

Based on the EECs, we selected two *Asarum* samples, i.e., 24 (*A. heterotropoides*) and 32 (*A. sieboldii*), and analysed the extracts by LC-MS (Figure 2). A total of 888 compounds were identified (Table S4). By comparing samples 24 and 32, we screened 317 differentially accumulated metabolites (DAMs): 183 in leaves, 105 in roots, and 29 commonly accumulated metabolites (Figure 3A,B). Within *A. heterotropoides,* the relative percentage content of 32 major compounds exhibited marked differences. Notably, 3,4-dimethoxycinnamic acid, DL-arginine, glutamic acid, (2E,4E)-N-(2-methylpropyl)deca-2,4-dienamide, N-desmethylvenlafaxine, acetyl-trans-resveratrol, L-pyroglutamic acid, (2E,4E)-N-(2-methylpropyl)dodeca-2,4-dienamide, 5-methyluridine, and several others were present in higher quantities in roots compared to leaves, whereas leaves had markedly higher contents of (3,4,5-trihydroxy-6-methyloxan-2-yl)2-(methylamino)benzoate, saxitoxin, 4-methoxycinnamaldehyde, salsolinol, choline, normorphine, and others (Table 1). The DAMs clearly separated the samples (Figure 3C). They were enriched in various KEGG pathways including phenylpropanoid, flavonoid, unsaturated fatty acid, and monoterpenoid biosynthesis and tyrosine, purine, galactose, and fatty acid metabolism (Figure 3D). Lipid mass annotation highlighted that the DAMs are classified as fatty acyls, glycerophospholipids, polyketides, prenol lipids, sphingolipids, and sterol lipids (Figure 3E). 

### 2.4. Pharmacodynamic Evaluation of Essential Oil and Ethanol Extracts of Asarum Roots

The results clearly showed that different doses of volatile oil extracts of *Asarum* roots significantly reduced the degree of ear swelling (Table 2). The degree of swelling decreased as the dose increased, i.e., from low to high. In the case of mice treated with EE, the degree of swelling generally decreased with increasing dose. The drug inhibition rate of EO ranged from 29.82% to 56.32%, whereas the drug inhibition rate of alcohol-soluble extract ranged from 17.72% to 38.60%. It was observed that under the same drug, the higher the dose, the higher the inhibition rate. In comparison, the inhibition rate of volatile oil extract was significantly higher than the inhibition rate of ethanol-soluble extract at the same dose level. 

The inhibition rate of drug concentration on acetic acid writhing in mice showed an effect in the order of E2_low_ < V2_low_ < E2_mild_ < E8_low_ < V2_mild_ < V8_low_ < ibuprofen < E2_high_ < E8_mild_ < V2_high_ < V8_mild_ < E8_high_ < V8_high_. The drug inhibition rate of the volatile oil ranged from 31.07% to 84.95%, and the drug inhibition rate of the alcohol-soluble extract ranged from 24.27% to 72.33%. For the same drug, the higher the dose, the higher the inhibition rate. At the same dose, the volatile oil extract showed a slightly higher inhibition rate than the alcohol-soluble extract (Table 3).

## 3. Discussion

*Asarum* species are rich in compounds with biological activities and are therefore used in TCM. Two species, i.e., *A. heterotropoides* and *A. sieboldii*, are commonly consumed as xixin in China. The content and composition of the EOs, EEs, and asarinin may differ depending on the drying method. We tested four drying methods, i.e., P1, P2, P3, and P4. Generally, the samples which were washed and then dried (shade, 30 °C, or 40 °C) had relatively higher EOC than those from which just soil was removed followed by drying (Table S2). The limited effect of these methods on EEC is a useful observation when considering large-scale drying and extraction with ethanol. Previously, air-dried methods have been reported to be useful for the study of EEs and identification of new phenanthrene derivatives [18]. The results that AsC was also lowest in P1 compared to the methods in which roots were washed prior to drying indicate the usefulness of the washing and drying. The non-significant effects of drying at 30 or 40 °C suggests just washing with water and shade drying is sufficient without the need of an oven as used in several other methods [19]. 

The fact that wild samples of *A. heterotropoides* had relatively higher EOC and lower EEC than cultivated samples is an important observation. Though, in this regard, limited knowledge is available in *Asarum* species, work on other species such as *Foeniculum vulgare* [20], *Thymus pannonicus* [21], *Salvia officinalis* [22], etc., has highlighted the existence of such differences. There could be several factors affecting this, such as the age of the plant, environment, genetic differences, etc., as reported in *Lavandula angustifolia* [23], *Cymbopgon martini* [24], and in *Asarum* species [19,25]. The results that samples from different growing regions of China showed content variation (Table 1) are consistent with earlier work that altitude can affect the metabolites of *Asarum* [19]. Further research on the effect of altitude between the same species under similar agronomic practices should be carried out to reveal the ideal altitude in relation to the location’s climate. Moreover, the difference between *A. heterotropoides* and *A. sieboldii* in terms of EOC, EEC, and AsC is understandable from a genetic differences point of view. This is consistent with the earlier reports that interspecific differences exist in terms of active compounds, e.g., in *Launaea* [26], *Ocimu* [27], and many others. Nevertheless, the highest contents of both EOC and EEC in *A. heterotropoides* indicates that it should be a preferred source of xixin when choosing between the two species.

The EO of *A. heterotropoides* has been previously explored for its composition, and >70 components have been reported [11]. Among them, 15 major components have been widely reported [12]. Haque, Moon, Saravana, Tilahun, and Chun [11] reported 44 EO components based on GC-MS; major components were 3,5-dimethoxytoluene, safrole, and myristicin. Similarly, Xiao et al. [28] reported 42 EO components by using micro-wave-assisted steam distillation followed by GC-MS. Our results that 78 compounds were identified are consistent with earlier work [12]. The presence of components with the highest proportions is also consistent with the previous works. The geographical variations were evident from the number of EO components. Work on different medicinal plants has revealed that geography can have significant effect on the EO content as well as composition [29]. Thus, we can conclude that *Asarum* species also exhibit EO content and compositional variations. Particularly, when considering the samples from the regions included in our work, Liuhe and Tonghua can be preferred. However, the sampling method, age of the plants, and other agronomic conditions should also be considered [19,25]. The observed higher quantities of safrole, elemicin, and myristicin in *A. heterotropoides* are noticeable variations compared to the higher presence of 3,5-dimethyoxytoluene in *A. sieboldii*. Thus, for their use as xixin, the relative content of each of the components should be given due consideration. Earlier work on *A. sieboldii* has shown the ten most important components of EO, including eucarvone, 3,5-dimethoxytoluene, isosafrole, methyl eugenol, and myristicin, together with five others [30]. However, our results based on EOC, EEC, asarinin, and six components of EO clearly indicate that *A. heterotropoides* is a better choice for xixin. 

The composition of the EEs showed a more diverse range of components compared to the EO (Figure 2). The 317 DAMs identified between the roots and leaves of *Asarum* species provide several reference metabolites for medicinal use. Consistent with our results, previous studies have reported the presence of lignans, monoterpenes, flavonoids, alkaloids, phenols, amino acids, and others [31]. The observation that both species had differences in EE content is consistent with the EEC results (Figure 1A; Table S2). Li, Cui, and Zheng [13] highlighted that the EE composition of *A. sieboldii* was different from other *Asarum* species including *A. heterotropoides* [18], *Asarum himalaicum* [32], *Asarum ichangse* [33], and members of the genus Aristolochiaceae [34]. However, the identification of 888 EE components is a valuable addition to the existing compound databases of the two species. The observation that several EE components were specific to each of the two species as well as in leaves or roots further proposes the specific uses of leaves and roots for targeted diseases. In this regard, the results of pharmacodynamic evaluation tests indicated that the EE as well as EO extracts significantly decreased the degree of swelling and increased inhibition rate of writhing in mice (Table 2 and Table 3). Although the EE showed a higher number of components, the increased drug inhibition rates of EO compared to EE highlights that the former is better. Most of the pharmacodynamic evaluation studies involving EE from *A. sieboldii* [35] and *A. heterotropoides* [36] have reported inhibition of writhing times of mice, increased pain threshold of thermal stimulation, and inhibition of xylene-treated ear swelling. As for EO of *A. seiboldii,* it has been reported that it could significantly reduce the amount of nasal secretions, sneezing, and the degree of nasal scratching in Sprague Dawley male rats [35]. *A. heterotropoides* EO can also induce antinociceptive and anti-inflammatory effects [36]. Several components of EOs from plants have been reported for their anti-inflammatory function via a range of metabolic regulatory pathways. For example, α-pinene, 3-carene, and limonene can regulate COX-2 expression and reduce inflammation [37]. Furthermore, EOs can also release anti-inflammatory mediators, which can regulate the expression of key genes related to reactive oxygen species scavenging, JAK/STAT signalling pathways, and interleukin and tumour necrosis, etc. [38,39]. Thus, our data are significantly important for choosing the *Asarum* variety and dose for future studies on characterization of individual EO components for their anti-inflammatory effects. 

## 4. Methods

### 4.1. Plant Material

A total of 36 *Asarum* samples were included in the study; Samples 1–31 were *Asarum heterotropoides* Fr. var. mandshuricum (Maxim.) Kitag, and Samples 32–36 were *Asarum sieboldii* Miq var. seoulense Nakai. Both species used in our study are sources of the traditional Chinese medicine xixin in the *Chinese Pharmacopoeia*. Samples were collected from Fusong, Jian, Liuhe, Tonghua, and Wangqing in Jilin Province, Xinbin in Liaoning Province, and Anguo in Hebei Province in China. Root and leaf samples were collected and then dried (Table 4; Figure S1). No permission is required to work on this species. Voucher specimens are available in the genebank herbarium of Jilin Agricultural University, China, under the number JINX760552-87. Official identification of the plant material was conducted by Prof Guanghui Wang.

All reagents were of analytical grade and were purchased from ThermoFisher Scientific, Dreieich, Germany; Shanghai Yuanye Bio-Technology Co., Ltd., Shanghai, China; Chengdu Alfa Biotechnology Co., Ltd., Chengdu, China; and Sichuan Weikeqi Biological Technology Co., Chengdu, China.

### 4.2. Sample Drying

*Asarum* roots were harvested and processed using four different methods. Each drying method included five replicates. In the first method, the soil was removed from the *Asarum* roots followed by drying in the shade for 7 days (P1). In the second method, the root samples were washed with water and dried in the shade for 7 days (P2). In the third method, the root samples were washed and dried for 48 h at 30 °C (P3). In the fourth method, the root samples were washed with water and dried for 48 h at 40 °C (P4).

### 4.3. Essential Oil Extraction and GC-MS Analysis

The EO in the root of *Asarum* was extracted by steam distillation. For extraction, the samples were ground on a stainless-steel mesh (40 mm, 0.014-inch wire diameter). The mesh samples (20 g) were hydrodistilled for three hours until no more oil was extracted according to the standard method of the *Chinese Pharmacopoeia* [40]. The EO was collected and stored at −20 °C. Then, 100 μL of EO was dried over anhydrous sodium sulphate and diluted 50 times with ethyl acetate. An aliquot (1 μL) of the sample was analysed by GC-MS using the HP-5 MS quartz capillary chromatographic column (30 m × 0.25 mm × 0.25 μm) (19091S-433UI, Agilent Technologies, Andover, MA, USA) on an Agilent 6890N-5973MSD gas chromatography–mass spectrometer (GC-MS, Agilent, MA, USA). The analytical conditions were as follows: 40 °C (for 2 min), increasing the temperature from 40 °C to 160 °C at a rate of 2.5 °C min^−1^, from 160 °C to 280 °C at a rate of 8 °C min^−1^, and finally maintaining a constant temperature of 280 °C for 10 min. The injection temperature was set at 280 °C. Helium was used as the carrier gas at a flow rate of 1.0 mL.min^−1^. An injection volume of 1 μL was used with a pulse without shunt. The mass spectrometer operated in an electron impact (EI) mode at 70 eV with scan ranges between 30 and 550 amu. The voltage of the multiplier tube was 0.98 kv. The ion source temperature was maintained at 230 °C and the quadrupole at 150 °C. A series of standard solutions were prepared by adding ethyl acetate to the mixed solution (1, 2, 5, 10, 20, 50, and 100 μg·mL^−1^). The injection volume was 1 μL, and the standard curve was plotted with the concentration as the abscissa (x) and the chromatographic peak area as the ordinate (y) (Table S1).

The total ion current chromatograms obtained were searched and compared with the spectrum in the NBS mass spectrometry database to determine the chemical components in the EO of each material.

### 4.4. Preparation of EEs and LC-MS Analysis

The leaf and root samples of the selected *Asarum* genotypes were ground into powder over a stainless-steel (ISO3310) mesh (#2 mesh in the *Chinese Pharmacopoeia*; Xinxiang Zhentai Mechanical Co., Ltd., Xinxiang, China) and weighed accurately. The EE was extracted by hot ethanol leaching. Briefly, the powdered sample (2~4 g) was placed in a 250 mL stoppered flask, and 100 mL of ethanol (95%) was added and weighed. The samples were extracted for one hour, then connected to a condensation tube and heated (100~150 °C) to boiling point for one hour. The extract was then allowed to cool to room temperature for one hour. After that, the weight loss was compensated by the addition of ethanol. A volume of 20 mL of the filtrate was placed in a dry and weighed evaporating dish (60 mm, Shengxing Chemistry Porcelain Factory, Tangshan, China) and evaporated in a water bath (HH-8, Yineng Changzhou, China). After evaporation, the extract was dried in a dryer (GZX-9070MBE, Boxun, Shanghai, China) for 30 min. The dried extract was dissolved in 2 mL of methanol, sealed, and stored in a freezer (−20 °C). 

Next, 100 μL of sample was taken into an Eppendorf tube, and 400 μL of 80% methanol was added and mixed by vortexing. The Eppendorf tube was kept in an ice bath for five minutes and centrifuged at 15,000× *g* at 4 °C for 20 min in a D3024R low-temperature centrifuge (Scilogex Company, Rocky Hill, CT, USA). The supernatant was collected and diluted with mass-spectrometry-grade water until the methanol content was 53%. The extract was then analysed on a Hyposil gold (C18) column (100 × 2.1 mm, 1.9 μm) (Thermo Fisher Company, Dreieich, Germany) and on an H-class ultra-high-performance liquid chromatograph (Waters Corporation, Milford, MA, USA). The analytical conditions were as follows. The column temperature and flow rate were 40 °C and 0.2 mL/min, respectively. Mobile phases A and B were water with 0.1% formic acid and methanol, respectively. Negative mode: mobile phase A was 5 mm ammonium acetate (pH = 9.0), and mobile phase B was methanol. Gradient elution procedure: 0 min: 98% (A) and 2% (B); 1.5 min: 98% (A) and 2% (B); 3 min: 0% (A) and 85% (B); 10 min: 0% (A) and 100% (B); 10.1 min: 98% (A) and 2% (B); 11 min: 98% (A) and 2% (B); 12 min: 98% (A) and 2% (B). The scan range was *m*/*z* 100–1500, and the ESI source settings were as follows. The spray voltage was 3.2 kV, the sheath gas flow rate was 40 arb, the aux gas flow rate was 10 arb, the capillary temp was 320 °C, the funnel RF level was 40, the aux gas heater temperature was 350 °C, and the polarity was positive, negative; the ESI source MS/MS secondary scan setting was data-dependent scans. 

### 4.5. Asarinin Extraction and Content Determination

Asarinin standard was prepared as a 1 mg.mL^−1^ solution with methanol. For sample preparation, the sample was passed through a stainless-steel mesh (No. 3 mesh, Xinxiang Zhentai Mechanical Co., Ltd., Xinxiang, China) and placed in a conical flask with a stopper containing 15 mL of methanol and weighed. Ultrasonic treatment (power 500 W, frequency 40 KHz) was then performed for 45 min, then the solution was cooled to room temperature. The loss of weight was made up with methanol. The solution was filtered through a 0.22 μm filter membrane (φ13, Jinteng, Tianjin, China), and the filtrate was injected directly into the UPLC system (Acquity UPLC H-Class, Waters, MA, USA). An Acquity C18 column (50 mm × 2.1 mm, 1.7 μm) (Waters, USA) was used for analysis.

The column temperature and flow rate were set to 30 °C and 0.3 mL.min^−1^, respectively. The detection wavelength was 287 nm. The injection volume was 2 μL. Gradient elution was performed using acetonitrile (A)–water (B) as the mobile phase: 0 min: 65% (A) and 35% (B); 7 min: 50% (A) and 50% (B); 10 min: 45% (A) and 55% (B); 12 min: 10% (A) and 90% (B); 15 min: 85% (A) and 15% (B); 18 min: 65% (A) and 35% (B). Asarinin standard was added with methanol to prepare a series of standard solutions (1, 5, 10, 50, 100, and 200 μg.mL^−1^), and the injection volume was 2 uL. The standard curve was plotted with the concentration as the abscissa (x) and the chromatographic peak area as the ordinate (y) in Table S1.

### 4.6. Study of the Anti-Inflammatory and Analgesic Effect of Asarum

The mice used in the experiments were purchased from Beijing Huafukang Biotechnology Co., Beijing, Ltd., China. The rearing room was designed to maintain environmental control for the animals, with a 12 h light–dark cycle, a temperature of 22 ± 2 °C, and a relative humidity of 50% ± 10%. The animals were allowed to eat and drink ad libitum during feeding. All animals were acclimated for three days prior to the main experiment. The animals were starved the night before the experiment and given unlimited access to water. The animal experiments were approved by the Animal Care and Use Committee of the Shanghai University of Traditional Chinese Medicine (Jilin Agricultural University; ethic number: 2023 02 02 001).

The mice were randomly divided into 14 groups of 10 mice each (Table 5). According to “Fang Pharmacology”, the dose for a 70 kg adult is 3 g, so the equivalent dose was calculated based on the body surface area [41]. The dexamethasone group received dexamethasone solution (0.260 g.kg^−1^). The extract was administered orally. The ear oedema test was performed as previously reported [42]. The degree of swelling (mg) and the inhibition rate (%) for each group were determined as previously reported [36]. In addition, the acetic acid writhing test was performed according to the method of [42,43].

### 4.7. Statistical Analyses

LC-MS data were processed and analysed as follows. Pearson’s correlation coefficient and statistical significance were calculated in R using the cor and cor.mtest packages, respectively (https://www.r-project.org/; accessed on 20 December 2023). The correlation was visualized using the corrplot package in R. The obtained total ion current chromatograms were searched and compared with the spectra in the mzCloud (https://www.mzcloud.org/; accessed on 20 December 2023), mzVault [44], and MassList (https://massbank.eu/MassBank/; accessed on 20 December 2023) mass spectrometry databases to determine the chemical components in the EEs. The differentially accumulated metabolites (DAMs) were screened if the variable importance of projection (VIP) > 1, *p* < 0.05, and fold change ≥2 and ≤0.5. Metabolites were annotated in the Kyoto Encyclopedia of Genes and Genomes (KEGG) database [45] and lipid maps [46]. The DAMs were enriched in KEGG pathways [47].

Data were analysed using a one-way analysis of variance in SPSS statistical software version 26.0. Tests of significant differences among treatments were analysed using the least significant difference (LSD) test. The significance level was set at *p* < 0.05 and 0.01.

## 5. Conclusions

Our results show that the active components in *Asarum* samples from different producing areas are different, i.e., EO, EE, and asarinin. There was a significant difference in EO components between *A. heterotropoides* and *A. sieboldii* samples. In general, wild and cultivated *Asarum* roots exhibit different EO, EE, and AsCs. Among the four tested methods of drying *Asarum* roots, washing the roots with water and drying (shade drying or drying at 30 or 40 °C) proved to be better than the method in which the samples were not washed and only the soil was removed prior to drying. Both species differ in terms of contents of six major EOs. Moreover, EEs of *A. heterotropoides* and *A. sieboldii* are different, hence can be used to distinguish both species. Our comparative results highlight that EOs have better pharmacodynamic performance compared to EEs. Thus, our results provide an important scientific basis for the cultivation, drying, and clinical application of *Asarum*.

## Figures and Tables

**Table 1:** Relative content differences in DAMs between leaves and roots of *A. heterotropoides*.

Compound	R24	L24
(3,4,5-Trihydroxy-6-methyloxan-2-yl)2-(methylamino)benzoate	0.1714	3.7265
3,4-Dimethoxycinnamic acid	4.7718	0.3927
2-(hydroxymethyl)-5-[6-(methylamino)purin-9-yl]oxolan-3-ol	4.2107	0.3638
Saxitoxin	0.1501	6.4897
4-Methoxycinnamaldehyde	2.4054	4.9084
[(2S)-2,3-dihydroxypropyl] (6Z,9Z,12Z)-octadeca-6,9,12-trienoate	0.1474	5.3676
Salsolinol	0.2827	2.7246
Isorhamnetin	0.0134	0.1739
Indoline-2-carboxylic acid	0.1248	0.6896
Choline	3.9046	4.8109
DL-Arginine	3.1928	0.577
6-Methoxyflavanone	0.1519	0.3365
Normorphine	0.1469	1.3902
(S)-Glutamic acid	2.9912	1.0074
(2E,4E)-N-(2-methylpropyl)deca-2,4-dienamide	4.4423	1.4944
Muramic acid	0.9621	0.0958
N-Desmethylvenlafaxine	1.0444	0.0757
gamma-Glutamylglutamic acid	2.3282	6.4897
Asarinin	1.5148	4.9084
O-Desmethylvenlafaxine	1.5097	4.8109
Asparagine	1.9377	3.7265
Sauchinone	2.103	3.6052
(9Z,11E,15Z)-13-hydroxyoctadeca-9,11,15-trienoic acid	1.0682	2.7246
9-Oxo-10(E),12(E)-octadecadienoic acid	1.2009	2.4944
Adenosine	1.1578	1.6364
Acetyl-trans-resveratrol	3.722	1.4944
L-Pyroglutamic acid	1.6079	1.3886
(2E,4E)-N-(2-methylpropyl)dodeca-2,4-dienamide	5.6764	1.2843
Methyl caffeate	1.0602	1.1626
5-Methyluridine	1.2054	1.0929
Menadione	1.1893	1.078
Kaempferol	0.0098	1.0608
Stiripentol	0.0889	1.0074
Aristolochic acids	0.0006	0.0062

**Table 2:** Effect of volatile oil and EEs of *Asarum* roots pretreatment on oedema degree and inhibition rate.

Group	Degree of Swelling (mg)	Inhibition Rate (%)	Group	Degree of Swelling (mg)	Inhibition Rate (%)
NS	11.40 ± 1.22	0	Ibuprofen	6.90 ± 0.43 **	39.47
V2_low_	8.60 ± 0.94 **	29.82	E2_low_	9.38 ± 0.53 *	17.72
V2_mild_	7.62 ± 0.99 **	33.04	E2_mild_	8.68 ± 0.92 **	23.86
V2_high_	6.52 ± 0.86 **	42.81	E2_high_	8.38 ± 0.88 **	26.49
V8_low_	6.42 ± 0.50 **	43.68	E8_low_	8.68 ± 0.76 **	23.86
V8_mild_	5.46 ± 0.78 **	52.11	E8_medium_	8.04 ± 0.61 **	29.47
V8_high_	4.98 ± 0.61 **	56.32	E8_high_	7.01 ± 0.80 **	38.6

NS = normal saline; V = essential oil extract; E = ethanol extract. The values are mean of n = 10 ± standard deviation. * = significant (*p* ≤ 0.05) and ** = significant (*p* ≤ 0.01).

**Table 3:** Effect of volatile oil and EEs of *Asarum* roots pretreatment on acetic acid writhing in mice.

Group	Number of Twists (Second Rate)	Inhibition Rate (%)	Group	Number of Twists (Second Rate)	Inhibition Rate (%)
NS	41.2 ± 2.94	0	DEX	19.6 ± 2.70 **	52.43
V2_low_	28.4 ± 3.20 **	31.07	E2_low_	31.2 ± 4.54 **	24.27
V2_mild_	21.8 ± 3.96 **	47.09	E2_mild_	26.6 ± 4.80 **	35.44
V2_high_	17.6 ± 1.81 **	57.28	E2_high_	19.2 ± 3.26 **	53.4
V8_low_	20.8 ± 3.27 **	49.51	E8_low_	25.8 ± 4.86 **	37.38
V8_mild_	14.4 ± 1.14 **	65.05	E8_medium_	18.6 ± 3.04 **	54.85
V8_high_	6.20 ± 2.58 **	84.95	E8_high_	11.4 ± 3.02 **	72.33

NS = normal saline; DEX = dexamethasone; V = essential oil extract; E = ethanol extract. The values are mean of n = 10 ± standard deviation. ** = significant (*p* ≤ 0.01).

**Table 4:** Details of the *Asarum* samples used in this study.

No.	Origin	Latitude and Longitude	Growth Years	Collection Date	No.	Origin	Latitude and Longitude	Growth Years	Collection Date
1	Fusong, Jilin	N 41°55′ E 128°01′	Four	2020.07	19	Liuhe, Jilin	N 42°07′ E 125°35′	Six	2020.08
2	Fusong, Jilin	N 41°55′ E 128°01′	Six	2020.07	20	Liuhe, Jilin	N 42°07′ E 125°35′	Eight	2020.08
3	Jian, Jilin	N 40°58′ E 126°18′	Seven	2020.06	21	Tonghua, Jilin	N 42°47′ E 126°23′	Four	2020.06
4	Jian, Jilin	N 40°58′ E 126°18′	Seven	2020.07	22	Tonghua, Jilin	N 42°47′ E 126°23′	Four	2020.07
5	Jian, Jilin	N 40°58′ E 126°18′	Seven	2020.08	23	Tonghua, Jilin	N 42°47′ E 126°23′	Four	2020.08
6	Jian, Jilin	N 40°58′ E 126°18′	Seven	2020.09	24	Tonghua, Jilin	N 42°47′ E 126°23′	Five	2020.07
7	Jian, Jilin	N 40°58′ E 126°18′	Wild	2020.06	25	Tonghua, Jilin	N 42°47′ E 126°23′	Five	2020.08
8	Jian, Jilin	N 40°58′ E 126°18′	Wild	2020.07	26	Tonghua, Jilin	N 42°47′ E 126°23′	Six	2020.06
9	Jian, Jilin	N 40°58′ E 126°18′	Wild	2020.08	27	Tonghua, Jilin	N 42°47′ E 126°23′	Six	2020.07
10	Jian, Jilin	N 40°58′ E 126°18′	Wild	2020.09	28	Tonghua, Jilin	N 42°47′ E 126°23′	Six	2020.08
11	Liuhe, Jilin	N 42°07′ E 125°35′	Four	2020.06	29	Wangqing, Jilin	N 43°36′ E 129°44′	Ten	2020.07
12	Liuhe, Jilin	N 42°07′ E 125°35′	Four	2020.07	30	Xinbin, Liaoning	N 41°22′ E 124°49′	Ten	2020.06
13	Liuhe, Jilin	N 42°07′ E 125°35′	Four	2020.08	31	Xinbin, Liaoning	N 41°22′ E 124°49′	Wild	2020.06
14	Liuhe, Jilin	N 42°07′ E 125°35′	Five	2020.06	32	Ningqiang, Shanxi	N 33°48′ E 105°31′	Five	2020.07
15	Liuhe, Jilin	N 42°07′ E 125°35′	Five	2020.07	33	Ningqiang, Shanxi	N 33°48′ E 105°31′	Six	2020.07
16	Liuhe, Jilin	N 42°07′ E 125°35′	Five	2020.08	34	Ningqiang, Shanxi	N 33°48′ E 105°31′	Wild	2020.07
17	Liuhe, Jilin	N 42°07′ E 125°35′	Six	2020.06	35	Lveyang, Shanxi	N 33°33′ E 106°16′	Six	2020.07
18	Liuhe, Jilin	N 42°07′ E 125°35′	Six	2020.07	36	Lveyang, Shanxi	N 33°33′ E 106°16′	Wild	2020.07

Note: Samples 1–31 are *Asarum heterotropoides* Fr. var. mandshuricum (Maxim.) Kitag (cultivated), and Samples 32–36 are *Asarum sieboldii* Miq var. seoulense Nakai (wild).

**Table 5:** Doses of volatile oil and ethanol extracts of *Asarum* roots used for pharmacodynamic study of mice.

Pretreatment Group		Dose (μL.kg^−1^)	Dose (μL.kg^−1^)
Normal Saline	NS	0	0
Volatile oil	V2 Low	1.65	1.65
Volatile oil	V2 mild	3.3	3.3
Volatile oil	V2 high	6.6	6.6
Volatile oil	V8 Low	1.7	1.7
Volatile oil	V8 mild	3.4	3.4
Volatile oil	V8 high	6.8	6.8
Dexamethasone/ibuprofen	DEX/IBP	(DEX) 0.26	(IBP) 0.104
Ethanol extract	E2 Low	8.85	8.85
Ethanol extract	E2 mild	17.7	17.7
Ethanol extract	E2 high	35.4	35.4
Ethanol extract	E8 Low	13.9	13.9
Ethanol extract	E8 mild	27.8	27.8
Ethanol extract	E8 high	55.6	55.6

DEX = dexamethasone; IBP = ibuprofen.

## Data Availability

The original contributions presented in the study are included in the article/Supplementary Materials, further inquiries can be directed to the corresponding authors.

## Supplementary Materials

The following supporting information can be downloaded at: https://www.mdpi.com/article/10.3390/ph17101301/s1, Figure S1. Map showing sites of *Asarum heterotropoides* and *Asarum sieboldii* samples from China. Table S1. Details of standard curve of each reference substance. Table S2. Biochemical profiles of *Asarum* roots processed by different methods. Table S3. List of constituents of essential oil detected in Asarum roots by using GC-MS. Table S4. List of components of ethanol extracts in *Asarum* roots (R) and leaves (L).
