# Supplementary figures and images for: Comparative Biochemical and Pharmacodynamic Analyses of Asarum heterotropoides Fr. Schmidt var. Mandshuricum (Maxim) Kitag and Asarum sieboldii Miq var. Seoulense Nakai Roots
Source: Pharmaceuticals (Basel). 2024 Sep 30;17(10):1301. doi: 10.3390/ph17101301 (PMC11509884; doi:10.3390/ph17101301)

## Slide 1
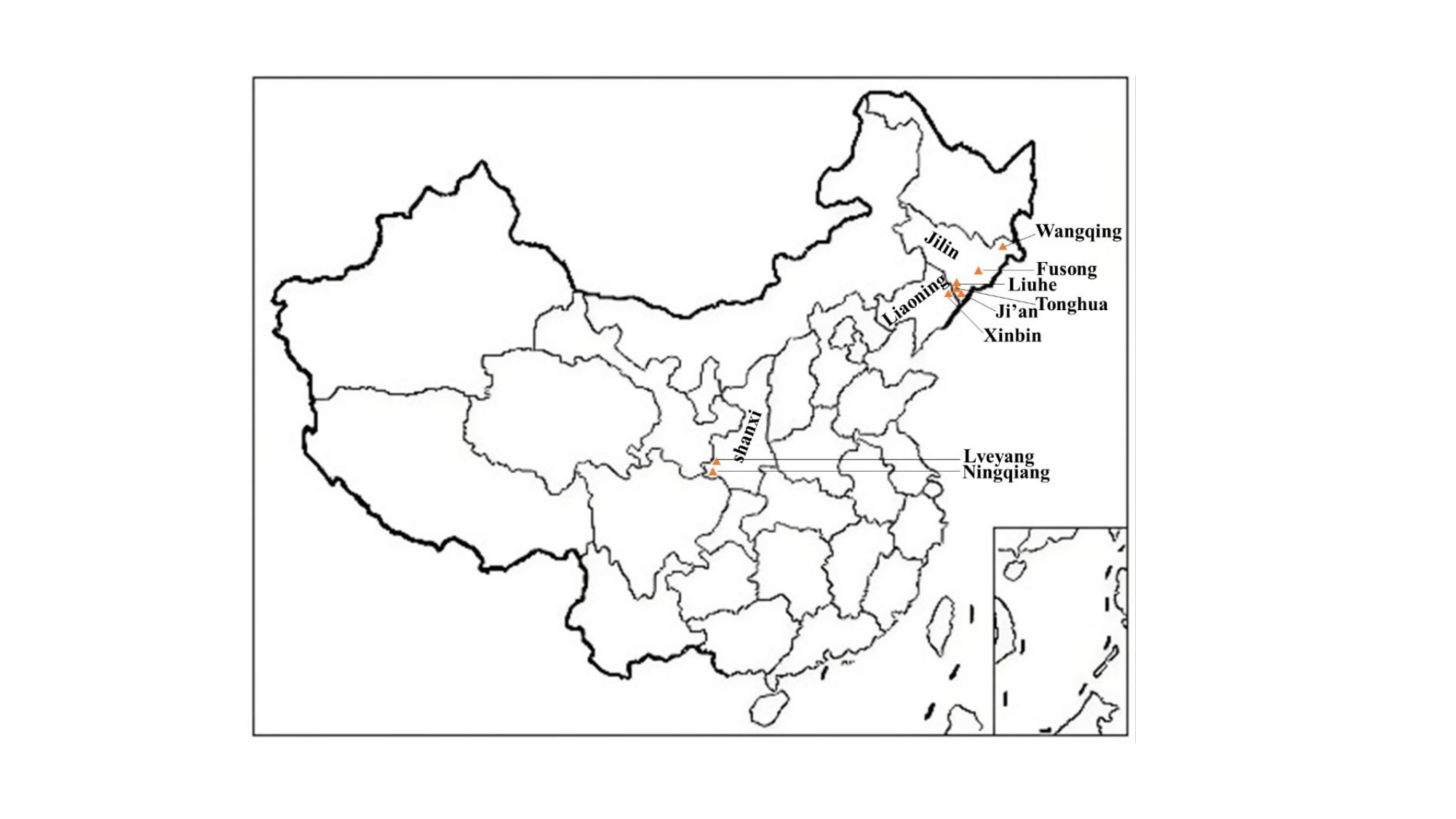

Supplement: Supplementary file 1 [file pharmaceuticals-17-01301-s001.zip › Figure S1 R1.pptx]
